# Cultured enterocytes internalise bacteria across their basolateral surface for, pathogen-inhibitable, trafficking to the apical compartment

**DOI:** 10.1038/srep17359

**Published:** 2015-11-27

**Authors:** Paul Dean, Sabine Quitard, David M. Bulmer, Andrew J. Roe, Brendan Kenny

**Affiliations:** 1 Institute for Cell and Molecular Biosciences, Newcastle University, Medical School, Newcastle-upon-Tyne, NE2 4HH, United Kingdom; 2 Institute of Infection, Immunity and Immunology, College of Medical, Veterinary and Life Sciences, University of Glasgow, Glasgow, United Kingdom

## Abstract

*In vitro*- and *in vivo*-polarised absorptive epithelia (enterocytes) are considered to be non-phagocytic towards bacteria with invasive pathogenic strains relying on virulence factors to ‘force’ entry. Here, we report a serendipitous discovery that questions these beliefs. Thus, we uncover in well-established models of human small (Caco-2; TC-7) and large (T84) intestinal enterocytes a polarization-dependent mechanism that can transfer millions of bacteria from the basal to apical compartment. Antibiotic-protection assays, confocal imaging and drug inhibitor data are consistent with a transcellular route in which internalized, basolateral-membrane enclosed bacteria are trafficked to and across the apical surface. Basal-to-apical transport of non-pathogenic bacteria (and inert beads) challenged the idea of pathogens relying on virulence factors to force entry. Indeed, studies with *Salmonella* demonstrated that it’s entry-forcing virulence factor (SPI-I) was not required to enter via the basolateral surface but to promote another virulence-associated event (intra-enterocyte accumulation).

Infection studies with non-polarisable epithelial cells, such as HeLa, helped to establish the concept of enterocytes being non-phagocytic towards bacteria—a premise supported by the discovery of pathogen-encoded virulence factors to force entry^1^,^2^,^3^. However, non-polarisable cells lack key features of enterocytes, such as absorptive microvilli, due to their inability to form tight junctional (TJ) complexes between neighbouring cells which provides ‘barrier’ (to limit the unregulated paracellular movement of ions, fluids and macromolecules) and ‘fence’ (to prevent the diffusion of membrane components between apical [lumen-facing] and basolateral [host-privileged surfaces]) functions^4^,^5^. Fence functionality is also critical for the targeted accumulation of cellular components to generate specialised features, such as, transporter-rich absorptive microvilli and enriching immune response-inducing antigen receptors on host-privileged surfaces. Enterocytes originate from crypt-located stem cells in a partially-polarised state with migration up the intestinal villus leading to the fully polarised/differentiated form^4^,^5^. While most epithelial cells differentiate into enterocytes others specialise to provide specific host-protective functions, including, mucus secretion (Goblet cells), releasing anti-microbial factors (Paneth cells) and sampling luminal contents (M-cells) for presentation to immune cells^4^,^5^. The physiological importance of epithelial barrier function in the gut is illustrated by the linkage of genetic-, environmental- and infection-related dysfunction to diarrhoeal, inflammatory and systemic disease^4^,^5^. Immortalised Caco-2 cells polarised on porous membranes (in Transwell inserts) provide a well-established model for enterocytes of the human small intestine that we have used to interrogate how a classic non-invasive enteric pathogen, enteropathogenic *E.coli* (EPEC), triggers disease-associated alterations^6^,^7^,^8^,^9^. Here, we describe how an unorthodox EPEC infection protocol revealed cultured enterocytes to have an unrecognised capacity to internalise bacteria (pathogens, non-pathogens and bacterial-sized beads) for transcellular translocation from the basolateral to apical compartment thereby challenging important beliefs about the biology of cultured enterocytes and invasive bacterial pathogens.

## Results

### Polarisation-dependent asymmetric translocation of *E.coli* across monolayers of cultured enterocytes

**Figure 1:**
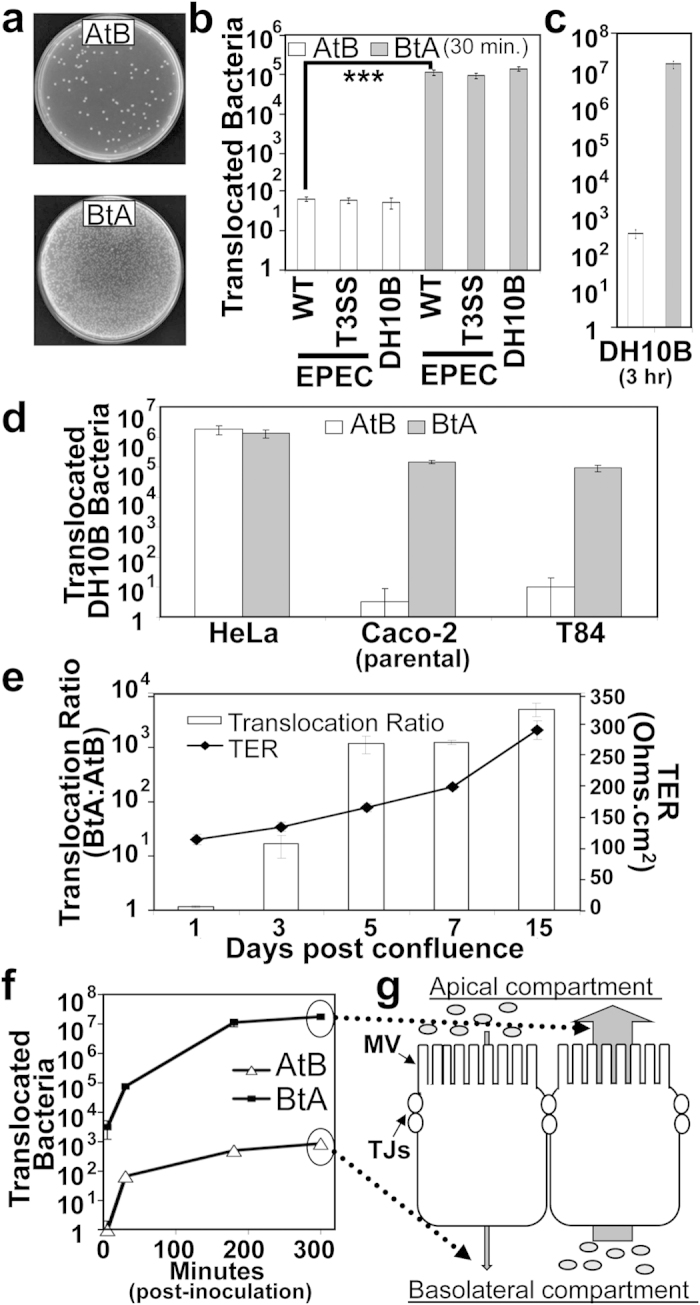
Basolateral-to-apical (BtA) translocation of *E.coli* across intact enterocyte monolayers. The apical or basolateral surfaces of non-polarisable (HeLa) or polarised (TC-7 and, in c, Caco-2 and T84) cell monolayers were inoculated with bacteria for 30 minutes—or indicated time—with media taken from the opposite compartment at indicated times for plating on agar (**a**) with the resulting counts plotted (logarithmic scale; (**b**–**f)**) as mean ± SD [error bars] from three independent experiments with *** indicating statistically significance (One-way ANOVA with Tukey Post Hoc Test) of p < 0.001. Monolayers were infected with enteropathogenic *E.coli* (EPEC)—wildtype (WT) or avirulent (T3SS-deficient) mutant—or innocuous K12 *E.coli* (DH10B). Polarised Caco-2/TC-7 and T84 cells are established models of human small and large intestinal enterocytes. AtB indicates infected apical surface taking samples from the basolateral well for enumerating bacterial numbers (opposite for BtA). The BtA:AtB ratio is provided alongside the Transepithelial Electrical Resistance (TER) value showing mean ± SD [error bars] from three independent experiments (**e**) with a time course study in (**f**). Note the error bars are often too small to detect. Graph illustration of data with MV and TJ indicating microvilli and tight junctions respectively (**g**).

Caco-2 enterocytes (polarised on membranes containing 3 μm pores in Transwell inserts) were infected with EPEC at the basolateral side to investigate whether interaction at this, normally inaccessible, surface would trigger disease-associated alterations. Unexpectedly, this work indicated that EPEC could access the opposite (apical) compartment in a manner independent of its main virulence factor—a Type Three Secretion Systems (T3SS) that transfer ‘effector’ proteins into enterocytes^6^. To further investigate this finding, a simple quantitative plating assay was used to monitor bacterial movement in the basolateral-to-apical (BtA) and apical-to-basolateral (AtB) directions (Fig. 1a). These assays involved a Caco-2 subclone, TC-7, to reduce possible issues from the recognised heterogeneity of cell types in the Caco-2 model^10^,^11^. As reported^12^, few EPEC (wildtype or avirulent T3SS-deficient mutant bacteria) translocated in the AtB direction with our data revealing significantly more (~2500 fold) wildtype EPEC (Fig. 1b; p = 0.006) translocating in the other (BtA) direction. Similar results were obtained with the T3SS mutant and non-pathogenic laboratory K12 (DH10B) *E.coli* strains (Fig. 1b) thereby uncoupling the translocation event from pathogen-encoded factors. Thus, further studies focused on DH10B where extended (3 hr) infections—which do not disrupt epithelia barrier functionality as assessed by transepithelial electrical resistance/TER measurements (Supplementary Figure S1 online)—revealed an even more dramatic asymmetric BtA:AtB translocation ratio (~40,000:1; Fig. 1c). Similar asymmetric translocation ratios were obtained for polarised Caco-2 and T84 cells (Fig. 1d); the latter a well-established model for enterocytes of the human large intestine^13^. The latter findings contrasted to symmetrical translocation profiles with confluent monolayers of HeLa cells (Fig. 1d) tentatively linking the asymmetric translocation process with the enterocyte polarisation/differentiation process. To interrogate the latter possibility studies were undertaken with TC-7 cells at different differentiation stages. As expected, infection of non-polarised TC-7 cells (1 day post-confluence; low TER value) led to symmetrical translocation ratios while differentiation—as evidenced by increasing TER values (Fig. 1e)—was associated with a developing asymmetrical bacterial translocation which was maximal for fully polarised (15 days post-confluence) cells (Fig. 1e). Time course infections revealed that few bacteria (~900) translocated in the AtB direction over a 5 hr infection period (Fig. 1f). By contrast, ~1000 and ~20,000 times more bacteria translocated in the BtA direction by 0.5 and 5 hr post-infection, respectively with ~18 million recovered from the apical compartment by experiment end (Fig. 1f,g). Thus, the enterocyte polarisation process provides a mechanism that enables host cells to translocate bacteria across the monolayer in a BtA-biased manner.

### BtA bacterial translocation via a transcellular route

**Figure 2:**
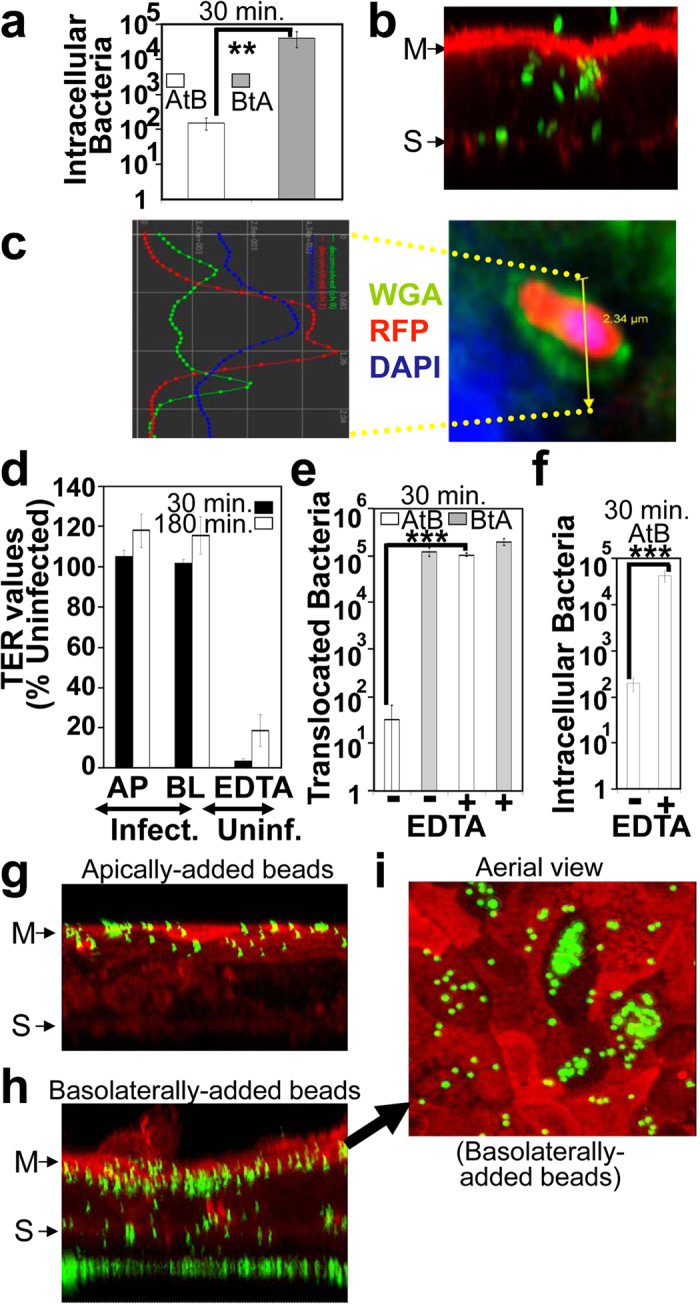
Transcellular bacterial translocation pathway. Polarised TC-7 cells were incubated with innocuous (DH10B) *E.coli* (**a**–**f**)—expressing EGFP (Green; **a**,**b**) or RFP (Red; (**c**))—or 1 μm fluorescent latex beads (Green; (**g**–**i**)). Monolayers (**d**–**f**) were pre-treated with (+) or without (−) 10 mM EDTA to disrupt barrier function—illustrated by decreasing Transepithelial Electric Resistance [TER] (**d**)—prior to infection. Numbers of translocating (**e**) or intracellular (**a**,**f**) bacteria is shown. Graphed data is mean ± SD [error bars] from three independent experiments with ** and *** indicating statistically significance (One-way ANOVA with Tukey Post Hoc Test) of p ≤ 0.01 and p ≤ 0.001 respectively. AtB indicates infected apical surface taking samples from the basolateral well for enumerating bacterial numbers (opposite for BtA). Monolayers (**b**,**c**,**g**–**i**) were fixed and stained for filamentous actin (Red; (**b**,**g**–**i**)) or DNA (host and bacterial, Blue; (**c**)). The basolateral surface was pre-labelled with FITC-conjugated wheat germ agglutinin (Green; (**c**)) prior to basolateral infection with confocal imaging signal intensity in the different channels along 2.34 μm yellow line shown: ‘Blue’ (bacterial DNA), ‘Red’ (bacterial RFP) and ‘Green’ (WGA-labelled basolateral membrane). Micrographs (**b**,**g**,**h**) are composites of 45–60 xy serial confocal-acquired sections (0.5 μm steps) with (**i**) an aerial view of the apical surface of monolayer shown in (**h**). M and S indicate position of cortical actin/microvillar region and Transwell porous support respectively (**b**,**g**,**h**).

As the TER data argued against disruption of cell-cell interactions (Supplementary Figure S1 online) which would enable paracellular translocation, studies focused on a transcellular route. Indeed, standard antibiotic (gentamicin) protection assays revealed that apical infections led to few intracellular bacteria (156+/−60) while basolateral infections resulted in significantly more (~258 fold; p < 0.01) intracellular bacteria (Fig. 2a). Importantly, confocal imaging of basolaterally-infected monolayers revealed *E.coli* beneath the support membrane, at the cell-substratum interface, above the actin-rich microvillus surface and, apparently, within enterocytes (Fig. 2b). To illustrate an intracellular location, the basolateral surface was labelled (using WGA-FITC) prior to basolateral infection, with confocal imaging revealing intracellular bacteria enclosed by FITC-labelled material (Fig. 2c). Given the clonal nature of the cell model it was surprising that <10^5^ bacteria (Fig. 2a) were recovered from a monolayer (~10^6^ enterocytes); but consistent with confocal imaging studies. The latter suggested that the transcellular translocation process involved a subset of cells or, more likely, that uptake was limited by the need for the bacteria (~0.5 × 2 μm in size expressing pili and/or flagella [1–3 μm]) to transit the 10 μm thick Transwell membrane via a limited number (~2 × 10^6^) of 3 μm pores in order to access the basolateral surface. To interrogate the latter possibility, monolayers were pre-treated with EDTA to disrupt cell-cell interactions with the expectation that this would provide apically-added bacteria greater (paracellular-mediated) access to the basolateral surface and, thus, increased internalisation levels. Indeed, EDTA-treatment disrupted epithelial barrier function (Fig. 2d) and while it had little impact on the number of BtA translocating bacteria (Fig. 2e) it dramatically increased the number of AtB translocating bacteria (>1000 fold; p = 0.001) with, as predicted, many more apically-added bacteria recovered from within cells (Fig. 2f; >100 fold; p < 0.001). However, the latter increase in intracellular bacterial numbers was surprisingly only to the level of standard basolateral infections (Fig. 2f versus 2a) suggesting that EDTA refractory cell-cell interactions continue to limit access of apically-added bacteria to the basolateral surface. In an additional strategy, enterocytes polarised on glass coverslips were physically ‘wounded’ before apically infecting with GFP-expressing DH10B and determining, via antibody labelling, if the cell-associated bacteria where intra- or extra-cellular. While enterocytes away from the wound sites had few cell-associated bacteria which were mostly extracellular (~80%; 962/1172) those close to the wound sites (Supplementary Figure S2 online) were mostly intracellular (~71%; 1143/1581). Replacement of bacteria for bacterial-sized fluorescent (1 μm) beads indicated that they were also internalised (Supplementary Figure S3 online) prompting imaging studies with cells polarised in Transwells. Such studies clearly revealed these inert particles to be substrates for internalisation into and across enterocytes, but only when added to the basolateral surface (Fig. 2g–i) supporting the findings with the bacteria. Collectively, the work reveals enterocytes have a polarisation-dependent capacity to internalise bacterial-sized particles which interact with their basolateral surface for transcellular translocation to the apical compartment.

### Transcellular trafficking of bacteria to and across the apical surface

**Figure 3:**
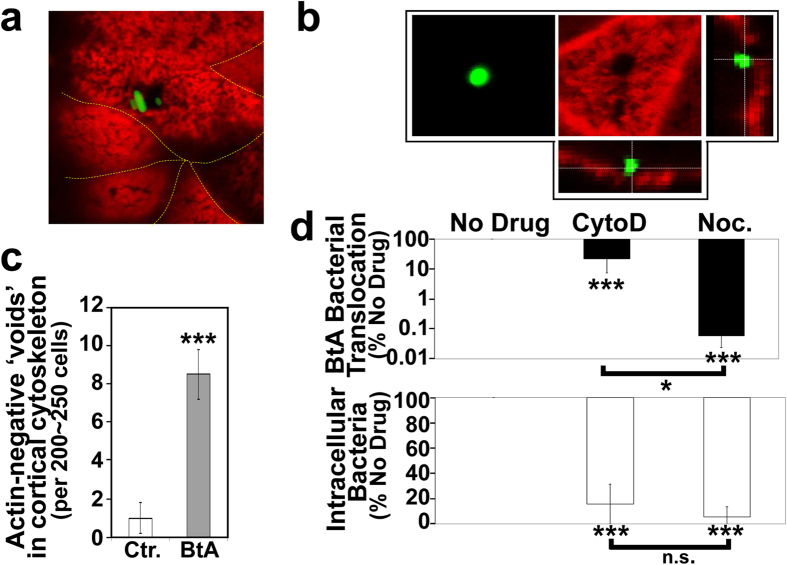
BtA bacterial translocation linked to cortical cytoskeletal rearrangement and transcellular trafficking events. Polarised TC-7 cells were infected at the basolateral surface with EGFP-expressing innocuous (DH10B) *E.coli* (Green). Monolayers (**a**–**c**) were fixed and stained for filamentous actin (Red) with confocal imaging revealing actin-negative ‘voids’ (**a**,**b**) that were quantified (**c**; data is mean ± SD [error bars] from three independent experiments, with *** indicating statistically significance (One-way ANOVA with Tukey Post Hoc Test) of p < 0.001). Micrographs (**a**, and larger panels in (**b**)) show xy-surface views with smaller panels (**b**) showing xz-sections. Ctr. indicates uninfected with BtA indicating that infected the basolateral surface (**c**). Monolayers (**d**) were left untreated (No Drug) or pre-treated with cytochalsin D (CytoD) or nocodazole (Noc) prior to infecting the basolateral surface with DH10B *E.coli* and enumerating the number of bacteria that reached the apical compartment (top panel) or were within (bottom panel) enterocytes. Data is mean ± SD [error bars] from 3 independent experiments shown as percentage recovered relative to No drug control (set at 100%) or between indicated data sets. * and *** indicating statistically significance (One-way ANOVA with Tukey Post Hoc Test) of p < 0.05 and p < 0.001, respectively compared to No drug control with n.s. indicating no significant difference.

Confocal imaging of the cortical actin—a recognised obstacle to trafficking events^14^ – unexpectedly revealed bacterial-sized ‘voids’—usually associated with bacteria (Fig. 3a,b)—that were rare in control (uninfected or apically-infected) monolayers with ~9 fold more for basolaterally-infected monolayers (Fig. 3c; p < 0.005). Thus, bacterial interaction with the basolateral surface appears to not only trigger uptake but also rearrangement of the cortical actin to enable internalised (membrane-enclosed) bacteria to be trafficked to the apical membrane for release into the apical compartment, presumably via an exocytosis-mediated event. A key role for the host actin and microtubule cytoskeletal networks in the uptake and BtA translocation processes was supported by dramatic reductions (Fig. 3d; p < 0.001) when monolayers were pre-incubated with inhibitors of vesicular-trafficking (nocodazole) or actin polymerisation (cytochalsinD) events. The smaller impact of cytochalsinD, compared to nocodazole (p = 0.043, on bacterial BtA translocation is associated with it disrupting epithelial barrier function (Supplementary Figure S4 online)—an event linked to enabling paracellular bacterial translocation (Fig. 2d, e). Thus, BtA translocation of bacteria across the enterocyte monolayer involves a transcellular route that, like the uptake process, depends on the cells retaining fully functional microtubule and actin networks.

### SPI-1 independent uptake and transcellular translocation of basolateral-infecting *Salmonella*

**Figure 4:**
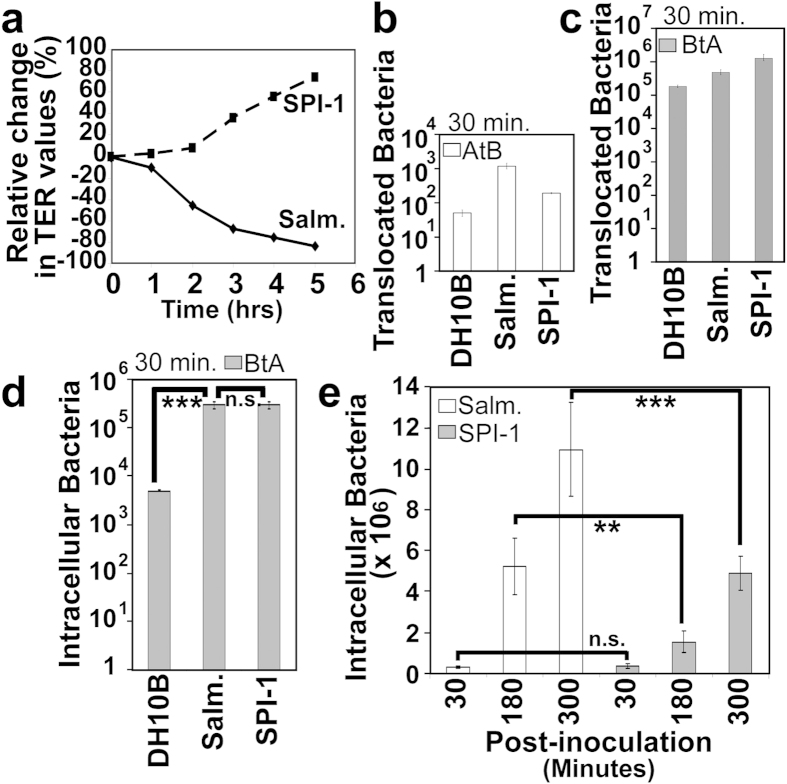
The *Salmonella* Typhimurium SPI-1 effector-delivery system is not required to enter enterocytes via the basolateral surface but promotes their intracellular accumulation. Polarised TC-7 cells were infected with *Salmonella* Typhimurium strains—wildtype [Salm.] or T3SS-deficient [SPI-1] mutant—or innocuous K12 (DH10B) *E.coli* at the apical (**a**,**b**) or basolateral (**c**–**e**) surfaces. Transepithelial Electrical Resistance (TER) changes were monitored (**a**) with bacterial translocation in AtB (**b**) or BtA (**c**) directions quantified following 30 min infections. AtB indicates infected apical surface taking samples from the basolateral well for enumerating bacterial numbers (opposite for BtA). Gentamicin-protection assays revealed the number of basolaterally-added bacteria that entered into enterocytes following 30 minute (**d**) or indicated (**e**) infection time periods. Data is mean ± SD [error bars] from three independent experiments with ** and *** indicating statistically significance (One-way ANOVA with Tukey Post Hoc Test) of p < 0.01 and p < 0.001 respectively with n.s. indicating no significant difference.

The uptake of innocuous *E.coli* (and bacterial-sized beads) across the basolateral surface questioned the belief that invasive pathogens rely on virulence factors to force entry. Thus, studies were carried out with a classic invasive enteric pathogen, *Salmonella enterica* serovar Typhimurium (*S.* Typhimurium) and an isogenic mutant that lacks the T3SS (SPI-1) linked to forced cellular entry^15^,^16^. Standard apical infections confirmed strain genotype since, as reported^16^, wildtype *S.* Typhimurium, but not the SPI-1 mutant disrupted epithelia barrier function (Fig. 4a), with the SPI-1 virulence factor playing a prominent role in enabling *Salmonella* to translocate in the AtB direction (Fig. 4b). Crucially, consistent with our data, SPI-1 functionality was found not to be required for basolaterally-added *Salmonella* to cross the monolayer (Fig. 4c) or to enter into the cells where significantly more were recovered compared to DH10B K12 *E.coli* (Fig. 4d; p = 0.006). Indeed, the BtA translocation data suggested that the SPI-1 mutant may translocate at greater, though not statistically significant (Fig. 4c; p = 0.6), numbers than the wildtype strain suggesting that SPI-1 functionality may inhibit the BtA translocation process and promote intra-cellularity. To investigate the latter possibility, time course studies were undertaken which revealed similar numbers of intracellular bacteria for both *Salmonella* strains following a short (30 min; p = 0.6) infection period with significantly fewer intracellular SPI-1 mutant bacteria at later—180 (p < 0.02) or 300 (p < 0.002) minute – post-infection time points (Fig. 4e). Thus, *Salmonella* does not require SPI-1 functionality to enter enterocytes via the basolateral surface but, by contrast, this virulence-critical factor promotes another virulence-associated event; intra-enterocyte accumulation.

## Discussion

Here we clearly show the polarisation process that generates well-established models for enterocytes of the human small (Caco-2; TC-7) and large (T84) intestine produces a transcellular transport pathway with a, hitherto unrecognised, capacity to transfer millions of bacteria from the basolateral to apical compartment. Such biology presumably remained cryptic due to pioneering infection studies—prior to the development of enterocyte models—with non-polarised cells establishing the concept of epithelia being non-phagocytic towards bacteria unless the bacteria encode virulence factors to force entry. Indeed, when polarisable epithelia were used such studies invariably involved i) pathogens that can force entry, ii) partially polarised cells—herein linked to low levels of BtA bacterial translocation, or iii) cells polarised on surfaces that only support apical infection. It is also possible that differences in reagents, cell models and/or experimental protocols contributed to a delay in appreciating the BtA bacterial transcellular translocation potential of cultured enterocytes. While AtB translocation was also evident, it was a much rarer event that probably relates to a paracellular translocation route. Our findings suggest that polarised Caco-2, TC-7 and T84 cells should be considered non-conventional phagocytes^17^ as they can internalise bacterial-sized particles across their basolateral surface for trafficking, not for intracellular degradation as per professional phagocytes, but to the opposite (apical) surface for expulsion. While, it is known that many invasive pathogens need to access (eg *Shigella flexneri*) or have a preference for (eg *Salmonella* Typhimurium and *Campylobacter jejuni*) the basolateral surface to enter into cultured enterocytes the uptake mechanism was assumed, until now, to depend on pathogen-encoded virulence factors^16^,^18^,^19^. Our findings raise the intriguing possibly that this polarisation-dependent biology may also be present in other polarisable epithelial, and perhaps endothelial, cell lines.

Despite the simplicity of our cultured model system—which lack specialised epithelial cells (stem, goblet, paneth, enteroendocrine, M-cells), underlying tissue and immune cells—it raises the provocative possibility that our findings may reflect an underappreciated existence of such polarisation-dependent biology in gut enterocytes. The latter hypothesis is not so unreasonable given increasing *in vivo* evidence for polarised epithelia being able to phagocytose larger particles (cells) while immune cells can move between and though endothelia^20^,^21^,^22^,^23^,^24^. Indeed, many invasive bacterial pathogens must, or can transit though M-cells *in vivo* to access the enterocyte basolateral surface for entry with one, *Salmonella* Typhimurium, recently shown to promote invasion by reprogramming adjacently-infected epithelia into M-cells^25^,^26^. Our findings may also help to explain why invasive enteric pathogens often target the basolateral surface *in vivo*—not only via M-cells but by disrupting cell-cell interactions or targeting the surface as cells slough-off 25—to engage the host-mediated uptake mechanism with virulence factors required, not to force entry but to inhibit expulsion via the BtA translocation pathway. We suggest that the BtA bacterial translocation pathway may be an unrecognised part of an enterocyte’s anti-infective functionality where it acts to provide a means to return into the gut lumen those rare bacteria that manage to access the basolateral surface—due to background ‘leakiness’ or disruption (through injury, infection or genetic defects)—while antigen receptor activation of immune responses initiate additional defensive measures. A phagocytic basolateral surface could also explain why disorders associated with bacteria crossing the gut barrier, such as inflammatory bowel disease (IBD), are linked with normally-harmless, non-invasive bacteria not only entering into the blood system but also into enterocytes^5^,^27^,^28^,^29^,^30^. Such BtA bacterial translocation biology, if verified, would undermine the linkage of IBD to an ‘invasive’ bacterial phenotype^31^,^32^,^33^—a correlation already questioned by the ability of a classic non-invasive pathogen (EPEC) to force entry into non-polarised cells^34^. Our findings should stimulate the undertaking of challenging live cell-imaging studies to interrogate the *in vivo* relevance of our discovery and to define the relationship of the uptake and/or BtA translocation processes to those described for the phagocytosis and/or BtA translocation of larger (epithelia)^20^,^21^,^22^,^23^,^24^ and smaller (viruses and sIgA protein complex) particles^35^,^36^.

In summary, our work clearly reveals an unrecognised property of cultured enterocytes extensively used in industry and academia^10^,^11^ with important implications for understanding the biology of invasive bacterial pathogens and enterocytes *in vitro* and *in vivo*.

## Methods

### Bacterial strains, plasmids and mammalian cells

Stains used were laboratory K12 *E.coli* (DH10B), enteropathogenic *E.coli* (EPEC E2348/69)—wildtype and T3SS-deficient (*cfm-*14) mutant^9^, *Salmonella enterica* serovar Typhimurium (*S.* Typhimurium SL1344)—wildtype and T3SS-deficient (SPI-1) mutant^37^—[kindly provided by Dr Anjam Khan, Newcastle University, UK]. A plasmid (pJ241) expressing a LacZ::RFP fusion protein (pJ241) was generated by cloning into a pUC-derived plasmid the synthesized DNA fragments (DNA 2.0) corresponding to the 192 bp region of the *E.coli lacZ* promoter and 726 bp of a codon-optimized derivative which encodes monomeric RFP^38^. The bacterial EFGP-expressing plasmid was from (Clontech). HeLa (ATCC, CCL-2), Caco-2 (ATCC, HTB-37), T84 (ATTC, CCL-248) and TC-7^39^ cells were cultured as described^8^,^9^.

### Bacterial Translocation and Invasion assays

Mammalian cells (seeded at ~100% confluence in Transwells inserts containing 3 μm pores; Corning) were routinely differentiated over 15 days, unless otherwise indicated, while HeLa cells were used 1 day post-confluence—as detach if left longer—with Transepithelial Electrical Resistance (TER) values measured as described^7^. Bacteria—often carrying an ampicillin resistance-encoding plasmid—were grown in Luria broth overnight at 37 °C (no shaking) and diluted 1:10 into Dulbecco’s Modified Eagle’s Medium (DMEM) for a hour (37 °C in 5% CO_2_ atmosphere) prior to adjusting the OD_600_ optical density to 0.1 (~1 × 10^8^ bacteria/ml) with 100 μl used for apical or basolateral infections. For routine 30 min. basolateral inoculations, Transwell inserts were inverted and inoculated 5 min. prior to removing the inoculums and re-inverting inserts into fresh DMEM. 20 μl aliquots were taken from basolateral and/or apical compartments (contain ~0.5 and ~1.5 ml DMEM respectively) as appropriate, for serial dilution plating onto agar plates with resulting colony forming units, CFU, enumerated using a colony counter (Scientific Lab Supplies). For time course experiments, the basolateral well was directly inoculated with 100 μl of bacterial suspension with sampled (for plating) apical volume replaced with DMEM. In some experiment EDTA (10 mM final conc.) was added apically (30 min.) to disrupt tight junctions between adjacent epithelia, prior to replacing with DMEM and initiating infection. For invasion assays, the apical and basolateral surfaces were washed (5 times with ice cold PBS) and incubated 1 hr with DMEM containing gentamicin (100 μg/ml final conc.)—to kill extracellular bacteria—washed and intracellular bacterial released (1% Triton-X100/PBS solution) for agar plate-mediated CFU enumeration. When appropriate, nocodazole (Sigma-Aldrich; 10 μM final conc.) or cytochalsinD (Sigma-Aldrich; 5 μM final conc.) were added to apical and basolateral compartments 3 hr prior to and during infection.

### Confocal and scanning electron microscopy

Transwell porous supports were excised and cell monolayers fixed (2.5% paraformaldehyde/PBS solution) prior to permeabilising (1% Triton X-100 in PBS) for staining filamentous actin (TRITC-phalloidin; Sigma-Aldrich) and DNA (DAPI; 4′,6-diamidino-2-phenylindole) as described^7^. In some experiments FITC-conjugated wheat germ agglutinin (Sigma-Aldrich) was used to label the enterocyte surface membranes, following manufacturer’s instructions, while 1 μm latex beads (polystyrene, fluorescent yellow-green; Sigma-Aldrich) were also used as uptake substrates. Cells were examined by confocal microscope using a Leica SP-2 confocal microscope as described^8^,^9^.

### Wounding assay

Cells polarised on 13 mm glass coverslips (VWR) were washed twice with DMEM and subjected to wounding (pulling a 10 μl pipette tip across the cell surface) prior to apical infection with GFP-expressing DH10B *E.coli* (MOI 50:1 for 3 h). Washed, paraformaldehyde-fixed cells were incubated with anti-*E.coli* antibodies (Abcam, AB13626) to label extracellular bacteria and visualised using ALEXA-conjugated secondary antibodies (Invitrogen, A21127).

### Statistical analyses

In all cases, unless otherwise stated, experiments were independently repeated 3 times. Data represents the mean ± SD. Significance within data sets were determined at 0.05 using a one-way ANOVA with a post-hoc Tukey test indicating significances between individual, or indicated data points.

## Additional Information

**How to cite this article**: Dean, P. *et al.* Cultured enterocytes internalise bacteria across their basolateral surface for, pathogen-inhibitable, trafficking to the apical compartment. *Sci. Rep.* **5**, 17359; doi: 10.1038/srep17359 (2015).

## Supplementary Material

Supplementary Information
