# Supplementary material for: Cultured enterocytes internalise bacteria across their basolateral surface for, pathogen-inhibitable, trafficking to the apical compartment
Source: Sci Rep. 2015 Nov 27;5:17359. doi: 10.1038/srep17359 (PMC4661573; doi:10.1038/srep17359)
Supplement: Supplementary Information [file srep17359-s1.pdf]

Cultured enterocytes internalise bacteria across their basolateral surface for, pathogen-inhibitable, trafficking to the apical compartment

Paul Dean[1], Sabine Quitard[1], David Bulmer[1], Andrew A. Roe[2] and Brendan Kenny[1*]

Supporting Figure Legends

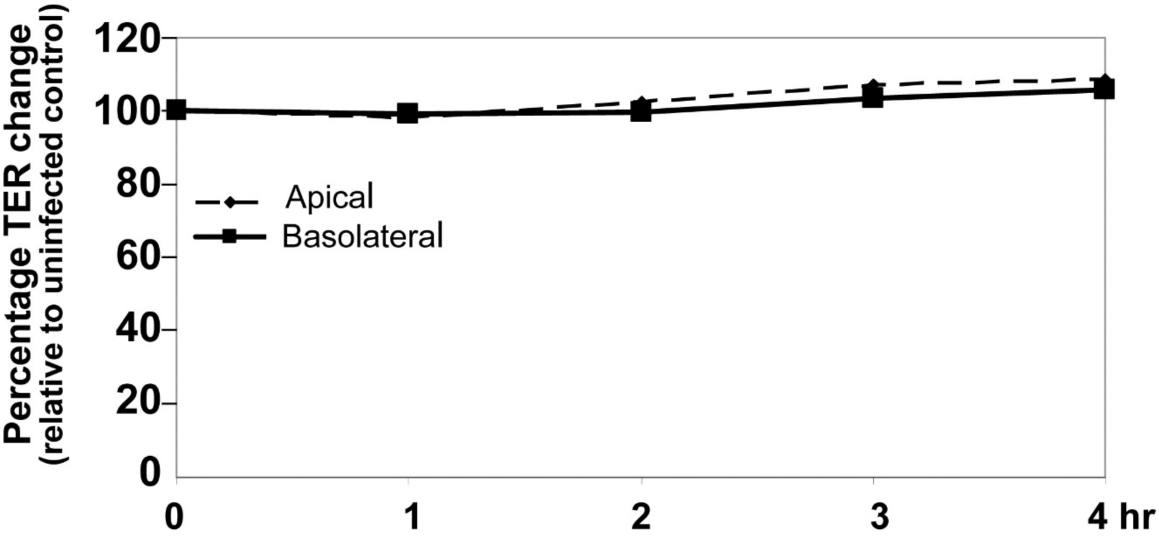

Fig. S1. Apical infection with innocuous *E.coli* does not disrupt epithelial barrier function.

Transepithelial electric resistance (TER) measurement on TC-7 cells polarised in Transwell inserts (containing 3µm pores) were taken before infection - background level; set as 100% - and at the indicated time points. Unbroken and dashed lines indicate data from monolayers infected with DH10B *E.coli* at the basolateral and apical compartments respectively.

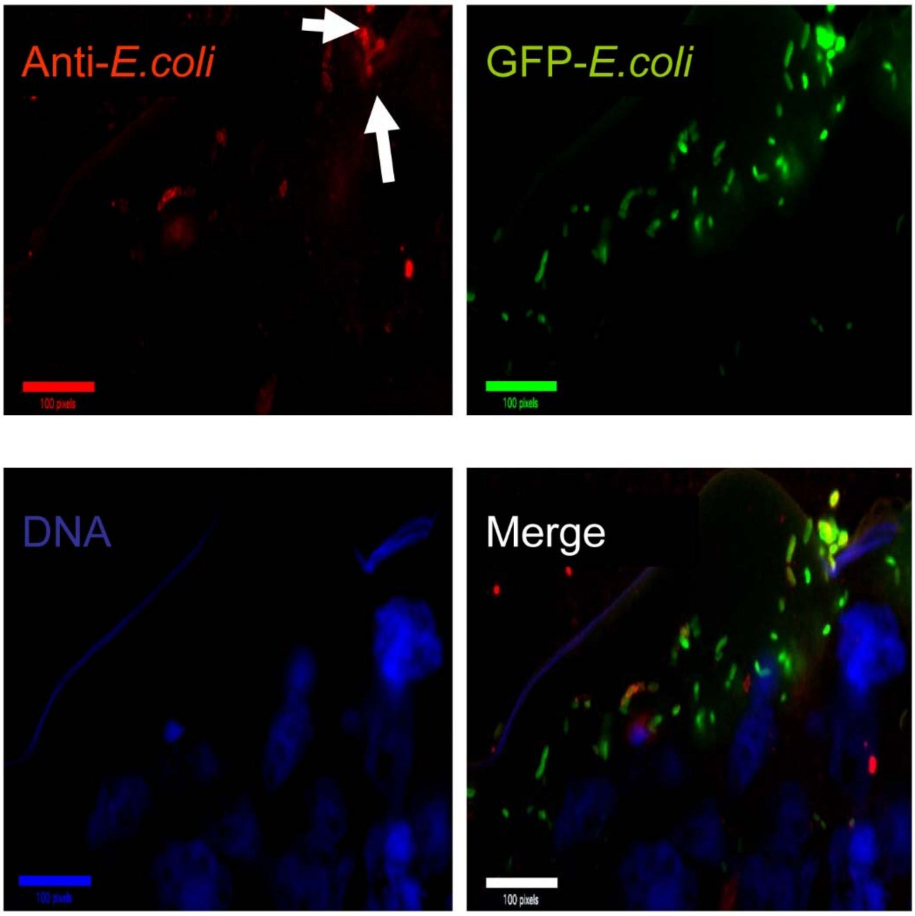

Fig. S2. Mechanical disruption of epithelial barrier function illustrates the capacity of enterocytes to internalise bacteria. TC-7 cells polarised on glass coverslips over 10 days were physically 'wounded' prior to apical addition with EGFP-expressing DH10B *E.coli* (Green). Post-fixing, monolayers were incubated with anti-*E.coli* antibodies to label extracellular (not intracellular, as intact host membrane excludes antibody) bacteria detected with appropriate fluorescent-labelled secondary antibodies (Red). Host and bacterial DNA were stained using DAPI (Blue). Arrows indicate extracellular bacteria (red and green) noting that most bacteria are solely green (i.e. intracellular).

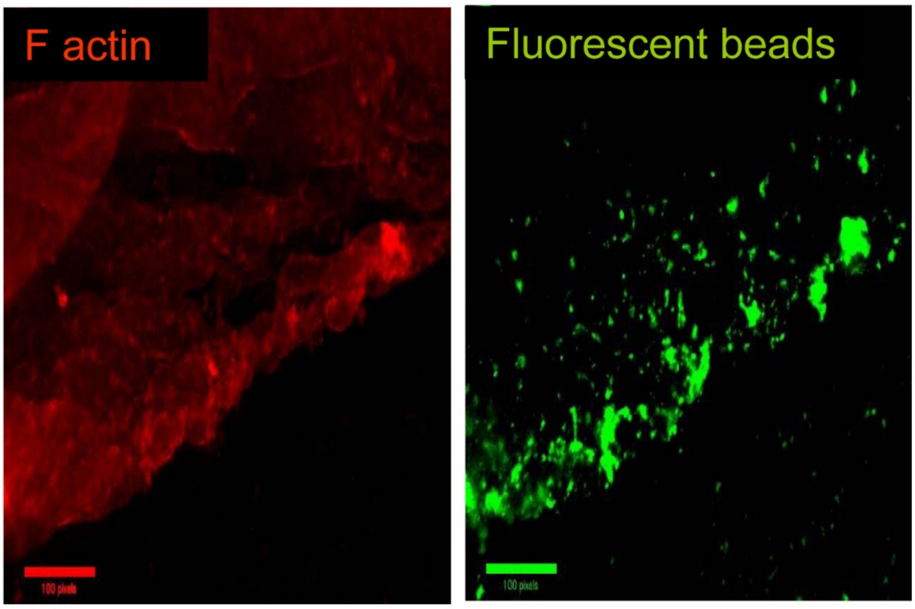

Fig. S3. Mechanical disruption of epithelial barrier suggests that enterocytes can also internalise 1µm latex beads. TC-7 cells polarised on glass coverslips over 10 days were physically 'wounded' prior to apical addition of 1µm fluorescently-labelled latex beads (Green). Post-fixing, monolayers were stained for filamenteous actin (Red) - to delineate host cells - with confocal imaging indicating the presence of beads within cells adjacent to the wound site.

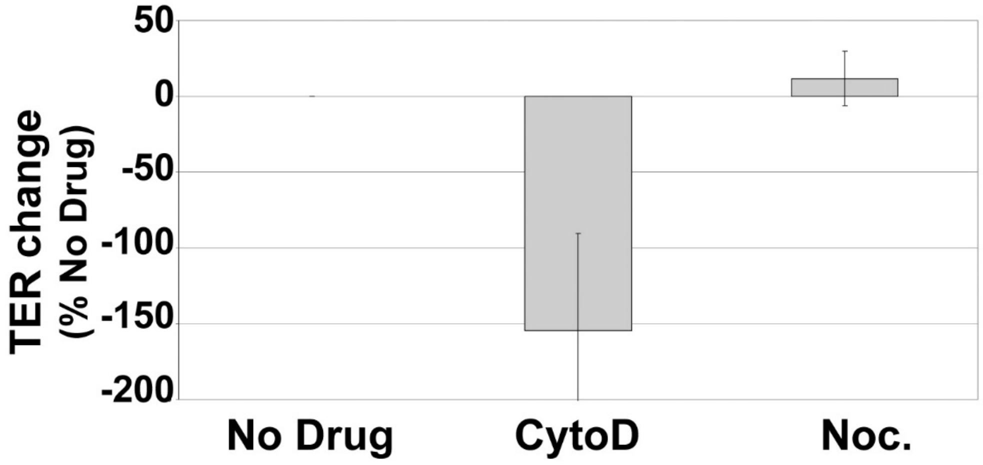

Fig. S4 Treatment of monolayers with Cytochalsin D, but not Nocodazole, disrupts epithelia barrier function. TC-7 cells polarised oin Transwell inserts (containing 3µm pores) were left untreated (No Drug) or pre-treated with Cytochalsin D (CytoD) or Nocodazole (Noc) prior to infecting (DH10B *E.coli*) with Transepithelial electrical resistance measurements taken before and after the infection period. Data (mean ± s.d. [error bars] from 3 independent experiments) is relative change in TER value compared to No Drug control data (set at 0%).
